# Setting hinge position distal to the proximal margin of the distal lateral femur reduces the maximum principal strains of the hinge area and risk of hinge fractures

**DOI:** 10.1002/jeo2.12015

**Published:** 2024-04-08

**Authors:** Atsuki Tanaka, Takehiko Matsushita, Tatsuya Nakatsuji, Yosuke Katsui, Kanto Nagai, Kyohei Nishida, Toshiji Mukai, Ryosuke Kuroda

**Affiliations:** ^1^ Department of Orthopaedic Surgery Kobe University Graduate School of Medicine Kobe Hyogo Japan; ^2^ Department of Mechanical Engineering Kobe University Graduate School of Engineering Kobe Hyogo Japan

**Keywords:** distal femoral osteotomy, finite element analysis, hinge fracture, hinge position

## Abstract

**Purpose:**

The optimal hinge position to prevent hinge fractures in medial closing wedge distal femoral osteotomy (MCWDFO) based on the biomechanical background has not yet been well examined. This study aimed to examine the appropriate hinge position in MCWDFO using finite element (FE) analysis to prevent hinge fractures.

**Methods:**

Computer‐aided design (CAD) models were created using composite replicate femurs. FE models of the MCWDFO with a 5° wedge were created with three different hinge positions: (A) 5 mm proximal to the proximal margin of the lateral epicondylar region, (B) proximal margin level and (C) 5 mm distal to the proximal margin level. The maximum and minimum principal strains in the cortical bone were calculated for each model. To validate the FE analysis, biomechanical tests were performed using composite replicate femurs with the same hinge position models as those in the FE analysis.

**Results:**

In the FE analysis, the maximum principal strains were in the order of Models A > B > C. The highest value of maximum principal strain was observed in the area proximal to the hinge. In the biomechanical test, hinge fractures occurred in the area proximal to the hinge in Models A and B, whereas the gap closed completely without hinge fractures in Model C. Fractures occurred in an area similar to where the highest maximal principal strain was observed in the FE analysis.

**Conclusion:**

Distal to the proximal margin of the lateral epicondylar region is an appropriate hinge position in MCWDFO to prevent hinge fractures.

**Level of Evidence:**

Level V.

**Abbreviations**
CAD: computer‐aided design
CT: computed tomography
DFO: distal femoral osteotomy
DICOM: digital imaging and communication in medicine
FEM: finite element method
LOWDFO: lateral opening wedge distal femoral osteotomy
MCWDFO: medial closing wedge distal femoral osteotomy
OA: osteoarthritis
PCF: pounds per cubic foot

## INTRODUCTION

Distal femoral osteotomy (DFO) is often performed to treat valgus knee alignment, lateral compartmental osteoarthritis (OA), cartilage injuries and patellar instability. An increasing number of studies have reported successful clinical outcomes, including high return to sports and work ratios, following DFO [1, 2, 4, 9, 19, 20]. Regarding surgical techniques, medial closing wedge DFO (MCWDFO) and lateral opening wedge DFO (LOWDFO) can be used without any significant differences in the final clinical outcomes [5]. MCWDFO is advantageous in patients who require large correction because it does not require bone grafting. Therefore, MCWDFO is a necessary surgical technique as DFO.

Although favourable outcomes can be expected after MCWDFO, complications associated with MCWDFO, such as nonunion, delayed union and hinge fractures, have been reported [6, 16, 22]. Among these, hinge fractures are relatively common complications during and after MCWDFO. The incidence of hinge fractures in previous reports was 50%–70% [7, 11, 14, 21]. Therefore, the prevention of hinge fractures is important for improving the outcomes of MCWDFO.

Owing to the high incidence rates of hinge fractures in MCWDFO, recent studies examined factors associated with hinge fractures and suggested that hinge position is one of the most important factors; Kim et al. reported that the upper border of the lateral femoral condyle could be recommended as an ideal radiographic hinge position to prevent unstable lateral hinge fractures during MCWDFO based on soft tissue coverage and bone density [10]. In addition, supra‐condylar hinge positions are associated with hinge fractures after MCWDFO [10]. Although these studies suggested that hinge position is the primary factor causing hinge fractures, the optimal hinge position for MCWDFO based on the biomechanical background has not yet been well examined.

In MCWDFO, the opposite lateral cortical area needs to be deformed during the closure of the gap without separation of the cortex, a so‐called fracture. From a biomechanical viewpoint, a higher strain on the cortex would increase the risk of fracture; therefore, a hinge position with a lower strain would be better suited for preventing fracture. Although conducting biomechanical tests with various hinge positions is preferable for determining the effects of the positions, the results can be inconsistent and time‐consuming. The finite element method (FEM) is useful for simulating various conditions and their effects in experimental computation models before conducting biomechanical tests or clinical applications. Previous studies have investigated the possible biomechanical effects of surgical techniques on the hinge during high tibial osteotomy using the FEM to improve surgical techniques, demonstrating the utility of the FEM [3, 12].

Therefore, the primary purpose of this study was to determine the optimal hinge position to reduce the risk of hinge fractures in MCWDFO from a biomechanical point of view using the FEM. The secondary objective was to examine whether the risk of hinge fracture was reduced when the optimal hinge position was set using an experimental MCWDFO model. We hypothesised that the optimal hinge position would be distal to the proximal margin of the lateral femoral condyle with low strain during gap closure and that the risk of hinge fracture would be reduced with the hinge position.

## METHODS

### Model development

**Figure 1:**
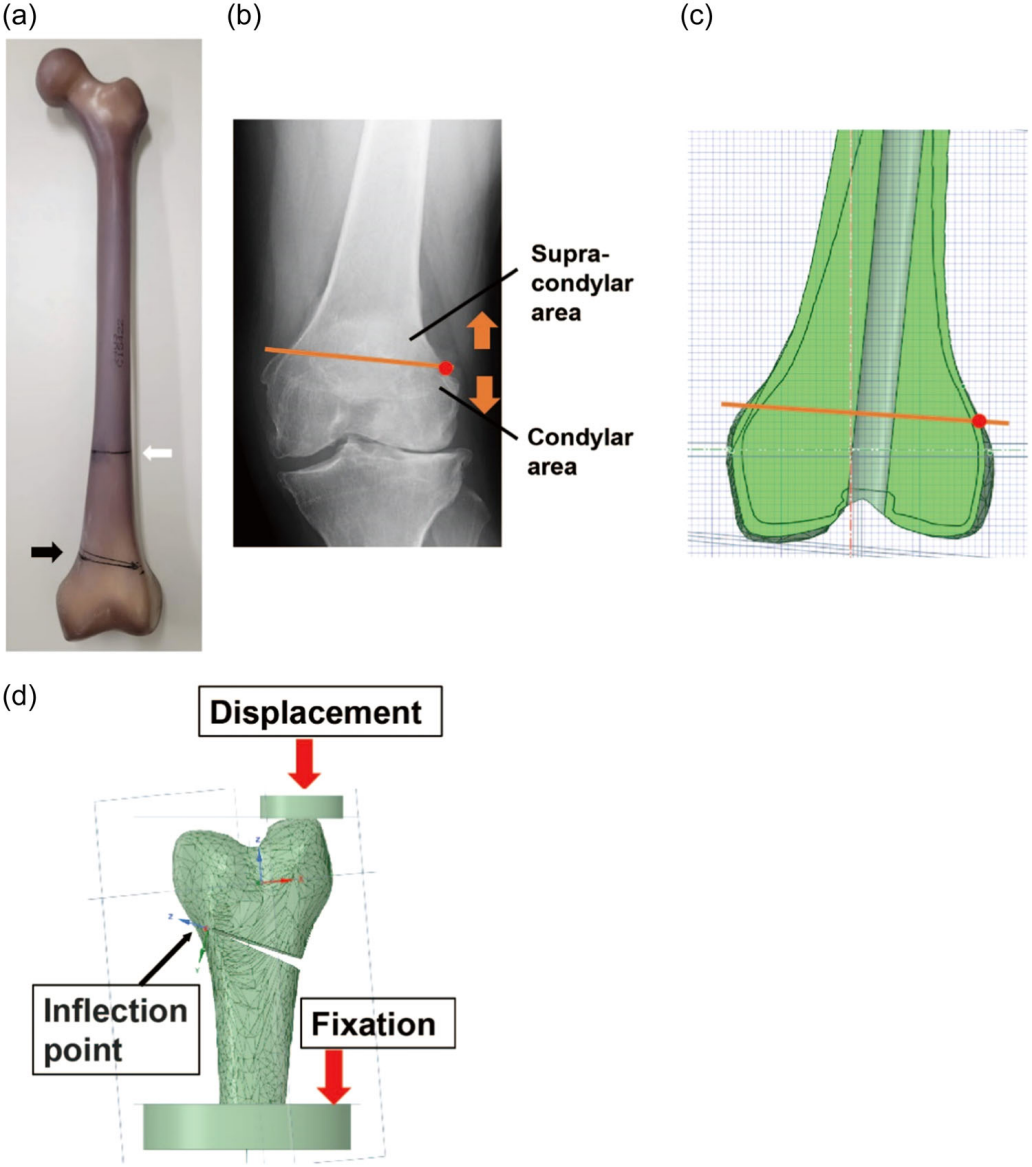
(a) The composite replicate the left femur. The white arrow indicates the cutting level at the distal one‐third shaft level. The black arrow indicates the osteotomy level and lines. (b) A plain radiograph anterior‐posterior view plain radiograph showing a patient with valgus knee osteoarthritis. The red point shows the inflection point of the lateral distal femur. The orange line connects the inflection points of the medial and lateral distal femur. The proximal and distal parts of the orange line were defined as the supra‐condylar and condylar areas, respectively. (c) A computer‐aided design (CAD) model of the composite femur. Cortical and cancellous bone were distinguished by differences in Hounsfield values. The inflection point of the lateral distal femur was determined in the anteroposterior plane at the distal femoral axis of the CAD model as the tuning point of the curvature. The red point shows the inflection point of the lateral distal femur. (d) Single‐plane medial closing wedge distal femoral osteotomy (MCWDFO) models set the wedge angle as 5°, and the hinge position was changed: (a) 5 mm proximal to the inflection point of the lateral distal femur, (b) at the inflection point, and (c) 5 mm distal to the inflection point.

The fourth‐generation composite replicate left femur bones of medium size (Sawbone, Pacific Research Lab Vashon) were used to create an osteotomy model (Figure 1a). The composite replicate bone was designed to mimic the cadaveric femur structure, and the material properties of the femur cadaver were validated as a usable alternative to the cadaveric bone in mechanical tests in previous studies [8, 23, 24]. The composite bone was made using short fibre‐filled epoxy as the cortical bone and solid rigid polyurethane foam 17 pounds per cubic foot (PCF) as the cancellous bone. Composite replicate femurs were scanned using computed tomography (CT) (Aquilion 64; Toshiba Medical Systems), and images were constructed at 0.4 mm intervals. Digital imaging and communication in medicine (DICOM) data were transported to the image processing software Mimics (version 22.0; Materialise) to create a computer‐aided design (CAD) model composed of cortical and cancellous bone. Cortical and cancellous bones were distinguished by differences in their associated Hounsfield values. The CAD model was exported to the three‐matic software (version 14.0; Materialise) and reconstructed smoothly by removing the rough part to create a more accurate femur model. The CAD model was transferred to the design three‐dimensional (3D)‐modelling software SpaceClaim (Canonsburg, ANSIS Inc. PA) to create the MCWDFO models. Then, the whole femur was cut 125 mm proximal to the most distal part of the medial condyle to reduce the computational effort. Basement and upper jigs were created and assembled to imitate the experimental test.

Single‐plane MCWDFO models were created from CAD models to examine the effects of hinge proximal‐distal levels. To mimic the operative situation, the hinge level was determined by setting the inflection point of the lateral distal femur, which can be identified under fluoroscopy, as the reference point, based on our clinical experience and previous studies [10] (Figure 1b). The inflection point of the lateral distal femur was determined in the anteroposterior view as the turning point of the curvature and was defined as the most proximal part of the lateral distal femur (Figure 1c). The hinge position was changed to (a) 5 mm proximal to the inflection point of the lateral distal femur (proximal model), (b) the level of the inflection point of the lateral distal femur (inflection point model), and (c) 5 mm distal to the inflection point of the lateral distal femur (distal model). The wedge angle was set to 5 ° for all three models.

### FEM

A linear tetrahedral finite element mesh was adopted, and homogeneous, isotropic, and linear elastic material properties were applied using the ANSYS software (Canonsburg, ANSIS Inc. PA) as follows: Young's modulus of 17 and 155 MPa for cortical and trabecular bone, respectively, and Poisson's ratio of 0.30 for both cortical and trabecular bones, according to a previous report [18]. Displacement was applied to the upper jig to close the gap after wedge removal, and the lower jig was fixed as the boundary condition (Figure 1d). Subsequently, a geometrically nonlinear analysis of the static structure was performed. To select the appropriate mesh size, six different mesh size models of the distal model (Model C) were created to conduct the mesh convergence test: 2, 1.5, 1.3, 1.1, 1, and 0.9 mm. The maximum principal strain of the cortical bone was calculated, and the percentage change in the maximum principal strain of the cortical bone between the 1 and 0.9 mm models was 0.5%. The percentage changes between the 1 and 1.1 mm models, 1 and 1.3 mm models, 1 and 1.5 mm models and 1 and 2 mm models were 2.4%, 4.6%, 7.8%and 8.9%, respectively. The 1 mm model was selected considering the calculation time. The numbers of nodes and elements in Models (A), (B) and (C) are listed in Table 1. The maximum principal strain (tensile) and minimum principal strain (compression) of the cortical bone at the hinge areas were calculated and compared among the models.

**Table 1:** The number of nodes and elements in Models A, B, and C.

	(A) Proximal model	(B) Inflection point model	(C) Distal model
Nodes	2,238,139	2,233,424	2,236,578
Elements	1,545,634	1,541,599	1,543,201

### Biomechanical tests

**Figure 2:**
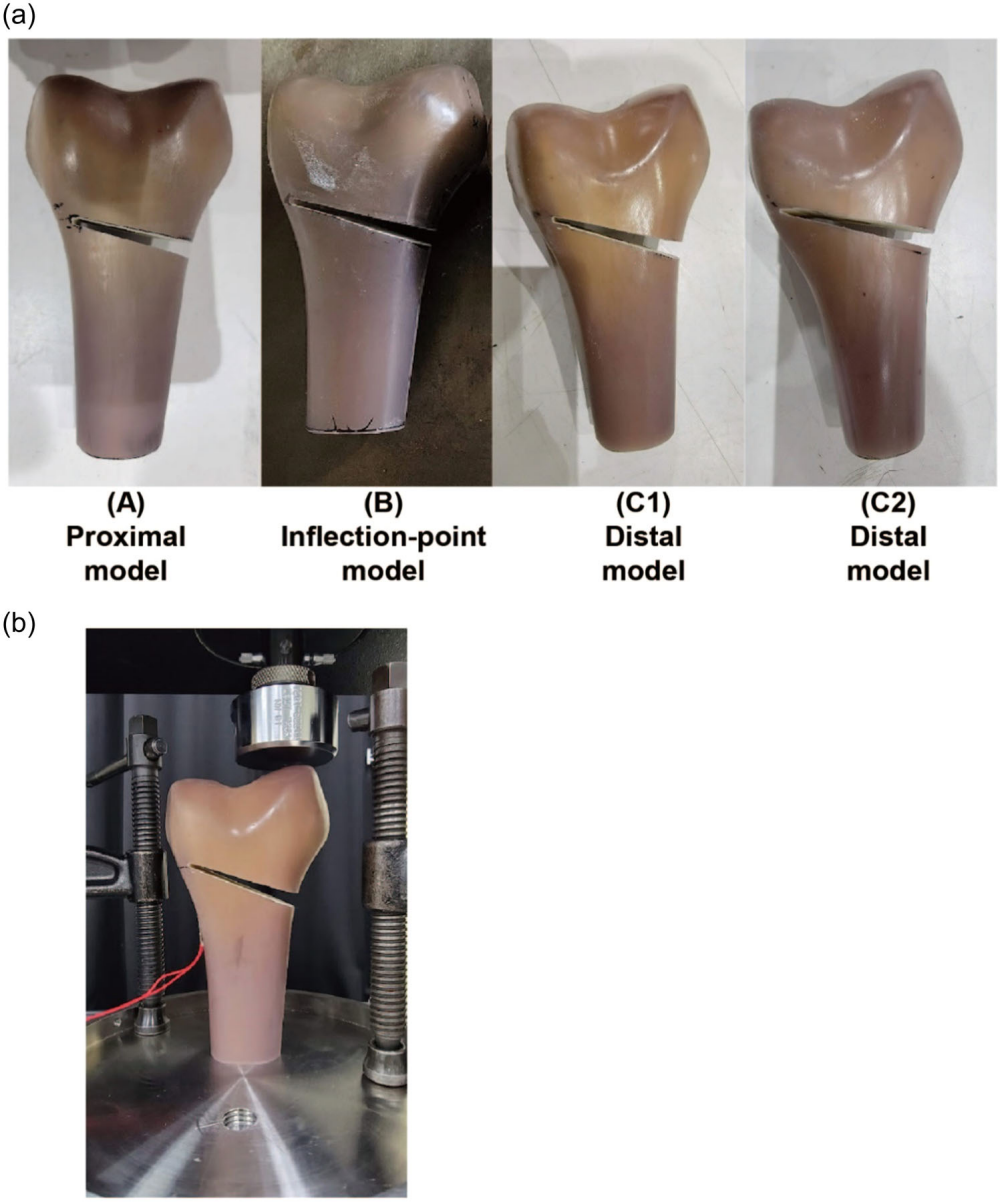
(a) The osteotomy models using composite replicate femurs. (A) Proximal model. (B) Inflection point models. (C1 and C2) Distal model. (b) The set‐upped composite femur (Model C1) for the biomechanical test. The vertical displacement with the same boundary conditions as the finite element (FE) models.

Biomechanical tests were performed using composite replicate femurs to validate the FE analysis. These models were developed under the same conditions as those used in the FE models. The proximal margin of the lateral condyle was determined anteriorly as the anterolateral corner of the lateral condyle. Each hinge position was confirmed by three testers. The 5 mm proximal and distal positions were determined using a ruler with a 1 mm scale and marked. The wedge angle was determined using an angle metre, and the length of the wedge width was measured. The composite replicate femurs were placed on a cutting machine (L‐300, LUXO, Aichi), and single‐plane MCWDFO was performed with the wedge angle set to 5°. After removing the wedge, the composite femur was cut at one‐third shaft level, and the distal part of the femur was used. Each of the (a) proximal and (b) inflection level models and two of the (c) distal models were created (Figure 2a).

The distal part of the composite bone was placed on the testing machine in an inverted position, as the distal part was above. A static compression testing machine (INSTRON 5567, Instron Corporation) was used to apply a vertical displacement with the same boundary conditions as the FE models (Figure 2b). To validate the FE analysis, the load‐displacement diagram of the experimental test for each model was compared with that of the FE analysis.

## RESULTS

### FE analysis

**Figure 3:**
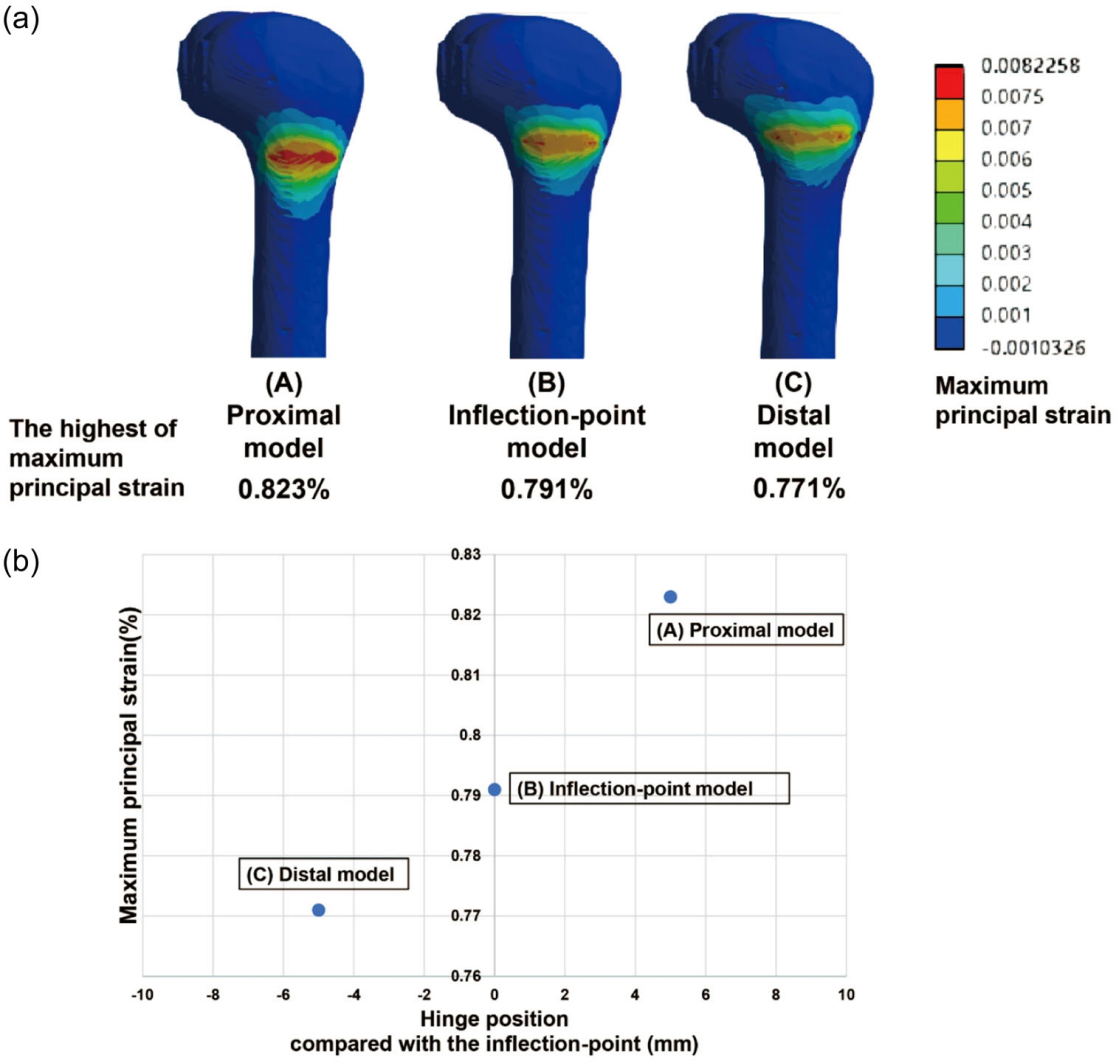
(a) The distribution of the highest value of the maximum principal strain when the wedge closed completely. The highest maximum principal strain was observed in the area just proximal to the hinge point. (b) The relationships between hinge position and the highest maximum principal. The highest maximum principal strains became smaller in the order of Models A > B > C.

The distribution of the maximum principal strain with the highest value at the complete closure of the wedge gap is shown in Figure 3a. The highest maximum principal strain was observed in the area proximal to the hinge. The highest maximum principal strains decreased in the order C > B > A (Figure 3b).

**Figure 4:**
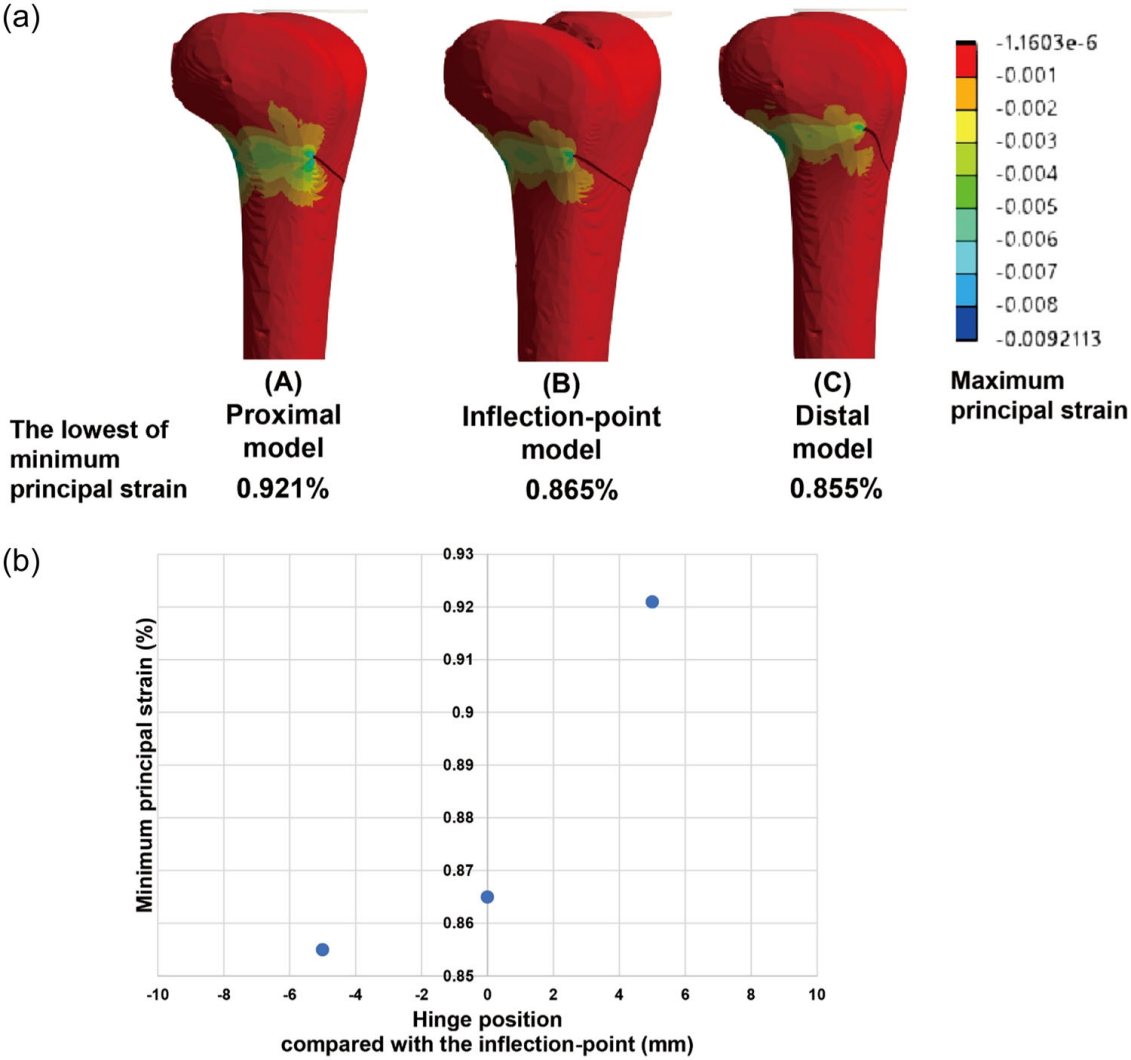
(a) The distribution of the lowest value of minimum principal strain when the wedge closed completely. The lowest minimum principal strain was observed in the area just proximally posterior to the hinge point. (b) The lowest minimum principal strains became smaller in the order of Models A < B < C.

The distribution of the minimum principal strain with the lowest value when the wedge gap is completely closed is shown in Figure 4a. The lowest minimum principal strain was observed in the area proximal to the hinge. The lowest minimum principal strains decreased in the order of Models A < B < C (Figure 4b).

### Biomechanical tests

**Figure 5:**
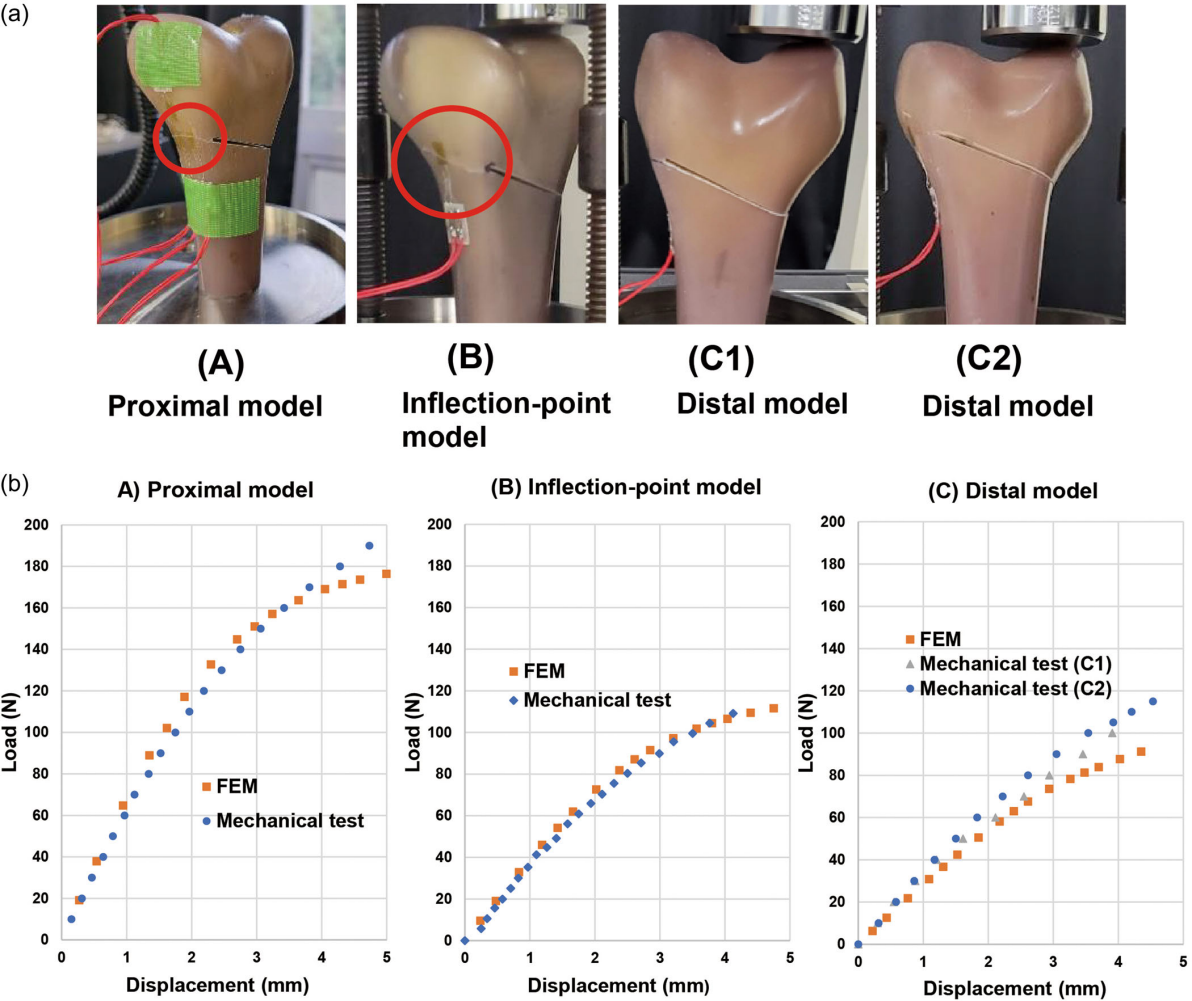
(a) Hinge fractures occurred in models (A) and (B), while the gap closed without hinge fractures in Models C1 and C2. (b) The load–displacement diagrams of the experimental test and the finite element analysis.

In the biomechanical tests, hinge fractures occurred in Models A and B. In contrast, no hinge fracture occurred in both C Models, and the gap closed completely (Figure 5a). In Models A and B, hinge fractures occurred when the wedge gap was closed. Fractures occurred in an area similar to where the highest maximal principal strain was observed in the FE analysis.

The load–displacement diagrams of the experimental test and FE analysis are shown in Figure 5b. The results of the FE analysis showed good agreement with the biomechanical tests.

## DISCUSSION

The main finding of this study was that the FE analysis demonstrated that the maximum principal strains were smallest in the hinge model, 5 mm distal to the proximal margin of the lateral distal femur (inflection point of the lateral condyle), compared with the proximal margin and 5 mm proximal levels. Moreover, hinge fractures occurred in the proximal margin and 5 mm proximal level models, whereas the gap closed completely without hinge fractures in the 5 mm distal model in the biomechanical test. Furthermore, the highest maximum principal strain was observed in the area proximal to the hinge position in all the models, and hinge fractures occurred in the area proximal to the hinge in the inflection and proximal hinge groups. These findings of the FE and biomechanical analyses suggest that 5 mm distal to the proximal margin of the lateral distal femur is an appropriate hinge position in MCWDFO to prevent hinge fractures.

Nha et al. performed a cadaveric study to examine the difference in the incidence of hinge fracture between the supracondylar hinge and condylar hinge MCWDFO [13]. However, the definition of the supracondylar and condylar regions has not been clearly described. They found a higher incidence of hinge fractures in the supracondylar hinge group than in the condylar hinge group (80% vs. 20%) [13]. In the study, the hinge position was set at 4­5 cm above the joint line in the supracondylar hinge group and 2–3 mm above the lateral epicondyle in the condylar group, which is similar to our current 5 mm‐distal‐hinge model. Accordingly, the same group reported that the lateral hinge position in the supracondylar area is significantly associated with the incidence of hinge fractures after MCWDFO in clinical cases [14]. Therefore, the position between the inflection point of the lateral condyle and the lateral epicondyle appears to be an appropriate hinge position to prevent lateral hinge fractures in MCWDFO.

Anatomical studies using cadavers have also been conducted, focusing on the possible role of the soft tissue surrounding the lateral condylar region in hinge support. Kim et al. performed an anatomical study using cadaveric knees and CT images. They found that the gastrocnemius attachment area and low‐bone‐density area were located near the proximal border of the lateral condyle [10]. They also performed MCWDFO on cadaveric knees and found that unstable lateral hinge fractures occurred more frequently when the hinge position was located outside rather than inside the femoral attachment of the gastrocnemius lateral head. Therefore, they recommend the proximal border of the lateral femoral condyle as the ideal hinge position based on soft tissue coverage and bone density. Oda et al. meticulously examined the lateral distal femur, focusing on the periosteum and joint capsule, using human cadavers [15]. They found that the thickness of the periosteum changed at the border region between the metaphysis and diaphysis, which can be determined as the turning point of the curve of the lateral distal femur on the radiograph, and markedly decreased in the diaphyseal region. Therefore, they concluded that the area distal to the proximal border of the metaphyseal region and the upper border of the lateral condyle, which can be determined under fluoroscopy, is the recommended hinge area, considering possible soft tissue support. Although the hinge areas recommended by the above two studies included a similar area, the proximal area of the recommended area appeared to be different, and the proximal area recommended by Oda et al. was more proximal to the proximal border of the lateral condyle [15]. Therefore, caution is necessary to utilise the reports when determining the hinge position.

Our study determined the optimal hinge position based on the hinge strain without soft tissue coverage, as closing the gap without cortical breakage seems to be the best‐suited approach rather than relying on the soft tissue. The present study showed that minimal strain was observed when the hinge position was set 5 mm distal to the inflection of the lateral condyle (proximal border of the lateral condyle) in the FE analysis. In addition, hinge fractures were not observed in the distal group, whereas displaced fractures were observed in the border and proximal groups. Similar to our results, Teo et al. recently reported that the occurrence rate of hinge fractures was reduced when the hinge point was set distal to the metaphyseal flare margin and gastrocnemius origin, which is similar to the area determined in our study [17]. Therefore, taken together with our results, the optimal hinge location appears to be distal to the proximal border of the lateral condyle.

### Limitations

This study had several limitations. First, the FE analysis and biomechanical tests were performed using only composite bones. In addition, biomechanical tests were not performed using cadaveric bones; therefore, the results may have been different if CT data from actual human patients or cadaveric bones were used. Second, all FE models used homogenous, linear isotropic elastic materials. Moreover, the presence of muscles was not simulated. It is conceivable that these muscles contribute to the prevention of hinge fractures. However, it is noteworthy that no hinge fractures occurred in the distal model composed of bone in the mechanical tests. Third, the distal hinge position was examined only 5 mm distal to the inflection point; other conditions were not examined. However, a distal position could not be established to avoid cutting the lateral condyle. Despite these limitations, the present study provides useful information on the optimal hinge position for MCWDFO to reduce the risk of hinge fractures.

## CONCLUSIONS

In the FE analysis, the maximum principal strain was minimal, and in the biomechanical tests, hinge fracture was not observed in the distal hinge model 5 mm distal to the proximal margin of the lateral condyle.

The appropriate hinge position in MCWDFO to prevent hinge fractures is distal to the proximal margin of the lateral condyle.

## AUTHOR CONTRIBUTIONS

Atsuki Tanaka was involved in the conception and design of the study, the acquisition, analysis and interpretation of the data, and writing the article. Takehiko Matsushita was involved in the conception and design of the study, development of the research, and writing the article. Tatsuya Nakatsuji, Yosuke Katsui, Kanto Nagai, Kyohei Nishida, Toshiji Mukai, Ryosuke Kuroda were involved in the acquisition and interpretation of the data. All of the authors were involved in the critical revisions of the article for its important intellectual content, and they all approved the final version of the article.

## CONFLICT OF INTEREST STATEMENT

Kanto Nagai is on the Editorial Board of this Journal. The other authors declare no conflict of interest.

## ETHICS STATEMENT

The requirement for ethical approval was waived as this study used no human or animal participants. Written informed consent was obtained from the patient.

## Data Availability

Data are available upon reasonable request.
